# Should anthropometric differences be considered when calculating the Rapid Shallow Breathing Index as a predictor of weaning outcomes in mechanically ventilated patients?

**DOI:** 10.62675/2965-2774.20250288

**Published:** 2025-07-15

**Authors:** Antuani Rafael Baptistella, Diego de Carvalho, João Rogério Nunes

**Affiliations:** 1 Universidade do Oeste de Santa Catarina Joaçaba SC Brazil Universidade do Oeste de Santa Catarina - Joaçaba (SC), Brazil.; 2 Hospital Universitário Santa Terezinha Joaçaba SC Brazil Hospital Universitário Santa Terezinha - Joaçaba (SC), Brazil.

Identifying the optimal time to extubate is critical, and predictive factors of extubation outcome may be useful for selecting patients ready for extubation, reducing the risk of reintubation, and improving their prognosis.^(1)^ The Rapid Shallow Breathing Index (RSBI) was proposed by Yang et al. in 1991^(2)^ and has since become the most widely used measure for predicting weaning and extubation outcomes.^(3)^

Despite its utility and importance, the RSBI does not account for anthropometric differences between patients. The RSBI divides the respiratory rate (RR) by the tidal volume (Vt), regardless of the patient's height or weight.^(2)^ Pulmonary Vt is determined by the predicted body weight (PBW), which is determined by the patient's height.^(4)^ Therefore, two patients with the same RR and the same Vt per kg could have different RSBIs, which could lead to different clinical decisions. In a hypothetical example, Patient 1, with an RR of 34 breaths/minute, 155cm tall, a PBW of 52.4kg, and a Vt of 6mL/kg (Vt = 314mL), has an RSBI of 108 breaths/minute/L, whereas Patient 2, with the same RR and Vt per kg, has a similar ventilatory status, but 185cm tall (PBW of 79.7kg and Vt of 478mL), has an RSBI of 71 breaths/minute/L, which is 34.3% lower than that of the first patient. Using the most common cutoff (RSBI < 105 breaths/minute/L^(5)^), the second patient would be extubated, whereas the first would not, even with the same RR and Vt per kg. If we analyze the RSBI of Patient 1, we may find a normal or overestimated RSBI and, conversely, an underestimated respiratory capacity. In case 2, we may have a normal or underestimated RSBI and consequently an overestimated respiratory capacity.

The accuracy of the RSBI was retrospectively evaluated in 308 extubated patients from three cohorts of a general adult intensive care unit (ICU), with data collected between 2017 and 2021 (ethical approval 4.793.240). The RSBI was measured with a ventilometer after 30 minutes^(6,7)^ of spontaneous breathing in the T-tube; the extubation criteria were based on the protocol published by Baptistella et al.,^(6)^ and patients who failed extubation were immediately reintubated. Patients were divided into two groups: those with central distribution heights and those with heights more than 1 standard deviation from the mean height. The receiver operating characteristic (ROC) curve was used to evaluate the ability to predict the extubation outcome at 48 hours in a dichotomous way. ^(8,9)^

Among the 308 extubated patients, 56.2% were male, the mean age was 60.2±17.2 years, 76.6% had a medical diagnosis, and the mean Acute Physiology and Chronic Health Evaluation (APACHE) II score at admission was 21.3 ± 7.4 (Table 1). Extubation was unsuccessful in 9% of the patients (n = 28) and successful in 91% (n = 280).

**Table 1:** Characteristics of patients with central heights and noncentral heights

		Total (308)	Central heights (204)	Noncentral heights (104)	p value
Age (years)		60.2 ± 17.2	59.7 ± 17.7	61.7 ± 16.2	0.342
Sex					
	Female	136 (44.2)	84 (61.8)	52 (38.2)	0.147
	Male	172 (55.8)	120 (69.8)	52 (30.2)	
APACHE II score (points)		21.3 ± 7.4	21.2 ± 7.6	21.4 ± 7.2	0.889
Days on MV		5.6 ± 3.2	5.6 ± 3.3	5.7 ± 3.1	0.978
Heart failure		34 (11)	21 (10.3)	13 (12.5)	0.568
COPD		50 (16.2)	33 (16.2)	17 (16.3)	1.000
Neurocritical		39 (12.7)	27 (13.2)	12 (11.5)	0.721
ICU outcome					
	Discharge	280 (90.9)	183 (65.4)	97 (34.6)	0.403
	Death	28 (9.1)	21 (75.0)	7 (25.0)	

APACHE - Acute Physiology and Chronic Health Evaluation; COPD - chronic obstructive pulmonary disease; MV - mechanical ventilation; ICU - intensive care unit. The results are expressed as the means ± standard deviations or n (%).

**Figure 1:**
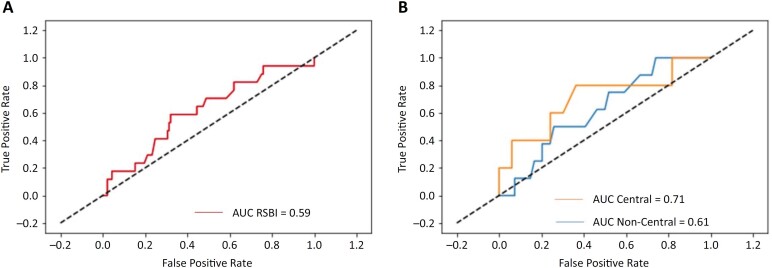
Receiver operating characteristic curve for evaluating the capacity of the Rapid Shallow Breathing Index to predict extubation outcomes within 48 hours.

The mean RSBI for patients who failed extubation was 50.2 ± 18.9, and for those who succeeded, it was 45.8 ± 18.2, with an area under the curve (AUC) of 0.59 (Figure 1A). However, when we separated patients with central distribution heights from those with more than 1 standard deviation from the mean height, we observed an important difference between the two groups (central heights AUC = 0.71 and noncentral heights AUC = 0.61; p < 0.0001 - Figure 1B), demonstrating the low predictive ability of the RSBI for patients who are not within the mean height. These two groups did not differ in terms of age, sex, APACHE II score, duration of mechanical ventilation, incidence of heart failure, incidence of chronic obstructive pulmonary disease, incidence of neurocritical disease or ICU outcome (Table 1).

A) Area under the curve for the Rapid Shallow Breathing Index (0.59). B) Area under the curve of the Rapid Shallow Breathing Index for central heights (0.71) and noncentral heights (0.61); p < 0,0001.

AUC - area under the curve; RSBI - Rapid Shallow Breathing Index.

Here, we showed that the RSBI has low accuracy in patients who are not within the average height range, which may help explain the low sensitivity and specificity recently described.^(5)^ The difference in accuracy obtained by the RSBI in patients with central height can be explained by the linear correlation between height and lung volume shown by Hepper et al.,^(10)^ which is not considered by the index.

As limitations, the calculation does not consider the sex of the patient, with average height differences affecting the predicted body weight, or possible differences in the RSBI between populations, such as patients with cardiac, pulmonary, or neurological disease, among others, which limits the generalizability of the results. In addition, the low mean RSBI in patients who failed extubation may have been influenced by the fact that, according to the institutional weaning and extubation protocol, all patients who were extubated had an RSBI < 105 breaths/minute.

Finally, this study shows that RSBI has different accuracies depending on the anthropometric specificities of patients and proposes a discussion of its limitations and possible ways to improve its accuracy, especially for patients whose average anthropometric characteristics are not known.
